# Developing a real-world database for oncology: a descriptive analysis of breast cancer in Argentina

**DOI:** 10.3332/ecancer.2022.1435

**Published:** 2022-08-04

**Authors:** Guillermo Streich, Marcelo Blanco Villalba, Christian Cid, Guillermo F Bramuglia

**Affiliations:** 1 Department of Oncology, Hospital Militar, Av Luis Maria Campos 726, Ciudad Autónoma de Buenos Aires (CABA), Argentina; 2 Department of Oncology, Centro Médico Austral, Montevideo 955 CABA, Argentina; 3 Sociedad Argentina de Cancerología, CABA, Av Santa Fe 1171, Buenos Aires, Argentina; 4 Argenomics, Parque Empresarial Austral, Edificio Insignia M4 - Planta Baja - Av Sto My C Beliera 3025, Pilar, Buenos Aires, Argentina; 5 Fundación Investigar, Parque Empresarial Austral, Edificio Insignia M4 - Planta Baja - Av Sto My C Beliera 3025, Pilar, Buenos Aires, Argentina

**Keywords:** RWD, real-world data, database, oncology, breast cancer, big data

## Abstract

**Introduction:**

Registries based on Real-World Data (RWD) are those obtained outside of systematised and randomised clinical trials. They allow the collection of information from a large number of patients and enable the participation of a significant number of professionals. PrecisaXperta is a web platform developed for this purpose with more than 2 years of operation, parameterised for oncology. Its design allows the construction of an epidemiological database in real time and exportable for processing.

**Objective:**

To describe the characteristics and operation of this online data recording tool, explain how it was developed and analyse the quality of the information recorded, taking as an example the data obtained for breast cancer.

**Materials and methods:**

Physicians, computer scientists and data science analysts participated in the development. Patient data, history, educational level, diagnosis, staging, molecular markers, quality of life, types of treatments, progression and response, imaging, complications, adverse events are some of the fields included. Data treatment in terms of encryption, anonymisation, protection and validation is also explained. The selected breast cancer data for description were processed with medium-level statistical programmes, since the number required to apply Big Data engines is not yet available.

**Results:**

From a total of 6,892 solid tumours, 1,892 were breast cancer and 1,654 were selected that complied with a data set minimum elaborated ad hoc. Cases from 13 provinces showed a geolocation bias according to the place of practice of the professionals in the collaborative network. The predominant lack of data was detected in molecular markers (ki67) and correlativity in some lines of treatment. Inconsistencies in dates and therapeutic schemes were also detected. Data curation made it possible to exclude them. The age of the patients was 55.3 ± 11.88 years. At the time of diagnosis, the predominance was in stage I: 36.48% and II 30.06%, with positive hormone receptors in 1,424 (89.96%) cases. The predominant treatments were hormonal (61.54%) and target directed with 30.85% for HER2(+) and 39.14% for HER2(−) accompanied in most cases (85.9%) by some period of chemotherapy. Immunotherapy was much less represented (0.36%). Data were processed, homogenised, pooled and presented and made accessible in a form suitable for application to RWD analyses.

**Conclusions:**

PrecisaXperta fulfils this purpose of systematising the information to facilitate its loading with its simple and intuitive interface. From the analysis of the data obtained in breast cancer, it is clear that some fields should be mandatory in order to improve the quality of the information. The results describing the registered breast cancers give us a surface view of the affected population and prepare us to design future studies when we have local Big Data. This type of development, with continuous improvements and online results, will allow with its dissemination, that the participating professionals have information of what happens in the real world, having available in a democratic way, the epidemiology to be able to study, publish and investigate with these data.

## Introduction

Artificial Intelligence (AI) tools are evolving constantly and make it possible to broaden our understanding of multivariate phenomena. They are used in countless fields where the quantity of data is so great that it is impossible to analyse using traditional statistical methods. AI tools have also enabled the development of predictive models in multiple specialities. Researchers use AI tools in systematised databases to create predictive models for oncology, and due to its high incidence, breast cancer.

Real-world data (RWD), and its analogue in the search for medical evidence (real-world evidence) [1, 2], represent novel approaches. They make it possible for large numbers of professionals who agree to participate to contribute anonymised epidemiological data collected from their patients to a systematised database that organises, hierarchises and facilitates analysis of that data. Systematising data entry also makes it possible to determine data reliability of data and assess biases and the feasibility of obtaining relevant evidence [3, 4].

PrecisaXperta is a RWD acquisition tool that Fundación Investigar developed on an online platform using systematised data for oncological diseases. After more than 2 years in operation, the authors decided to assess the content of the data PrecisaXperta had collected by focusing on one disease. Moreover, considering the operations of the upload interface, this study analysed changes resulting in improved data quality.

### Objective

The objective of this study is to describe how the PrecisaXperta data recording platform was developed and how it operates. This study also aims to analyse the quality of the data collected and suggest recording method improvements using the information obtained about breast cancer data collected in Argentina between August 2020 and November 2021.

## Materials and methods

Creating a user-friendly interface was vital for encouraging professionals to commit to sharing epidemiological information on RWD. A first step was therefore to invite stakeholders; including data science analysts, oncologists and programmers; to serve as developers and create an easily accessible, systematised online oncology platform. The basic platform structure required patient data, history, tumour descriptions, staging and lines of treatment, response and progression, complications, images, other medications, quality of life and adverse events. Developers agreed during this multidisciplinary effort to include multiple or simple preloaded options to reduce the use of keyboards. After developers defined fields, data and upload methods, the platform was then launched by inviting specialists to voluntarily record anonymous data from their patients. Developers also agreed that the epidemiology data the group recorded would remain freely available for any participants to use as they wish.

Thirty-eight specialist physicians from 13 Argentinian provinces participated by providing this data. The data recorded on PrecisaXperta (for 6,892 patients at the deadline for this publication) included data for 1,892 patients with a breast cancer diagnosis. Of these, 1,654 were selected for this analysis as they met the ‘minimum data set’ criterion the authors defined. The descriptors recorded were age, weight, history, comorbidities, geolocation (the province where the patient lives), TNM staging at diagnosis, hormone receptors, molecular markers and type of treatment received.

High-prevalence solid tumours were chosen for the data quality analysis. Inclusion criteria for selecting cases to assess were those with a breast cancer diagnosis and a minimum recorded data set, as shown in the tables below. Cases that did not meet these criteria were excluded.

The case analysis was conducted using Infostat, a general-purpose statistical analysis software. Infostat will, as data volume grows, make it possible to use advanced statistical methods such as modelling and multivariate analysis.

## Results

The data recorded for the condition selected (breast cancer) is described to assess its quality. Table 1 shows anthropometric data for included patients. Average age was just over 55 years, meaning that most patients were postmenopausal. Average height and weight fell within an average body mass index (ABMI) of 25.2, which PrecisaXperta calculated automatically. Among study group patients, 11% had a history of obesity, which is valuable data as obesity correlates with some malignant tumours [5]. Assessments of patients found that the most common histories were diabetes, obesity and smoking.

**Figure 1.**
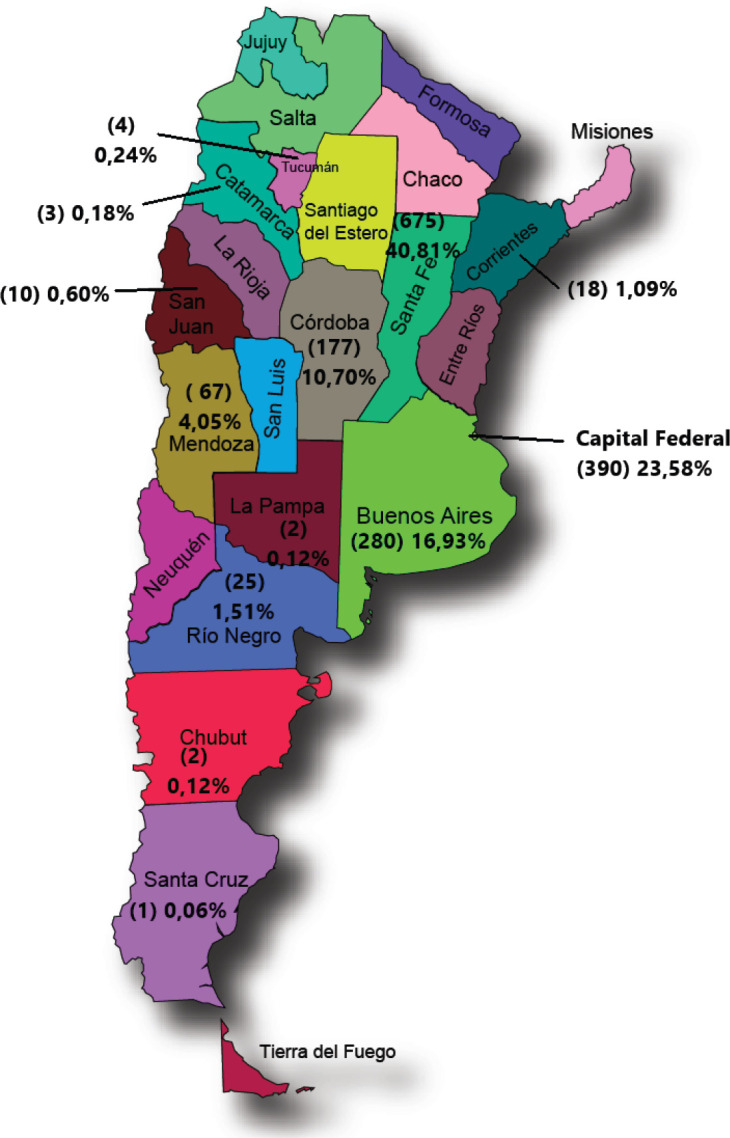
Patients recorded by residence (Province) N: 1,654.

The geographical origin of cases (Figure 1) showed larger proportions of cases that reflected provincial populations. Exceptions were Santa Fe, which was over-represented, and Buenos Aires which was underrepresented. Eighty percent of all cases were from three regions (Santa Fe, Buenos Aires and the Federal Capital), thus showing a representation bias stemming from the geographical distribution of the participating researchers.

Table 2 shows the number of patients by stage at breast cancer diagnosis. Nearly 100% of tumours had been staged in PrecisaXperta and the platform recorded the molecular markers of these patients. Classification by hormones and epidermal growth factor (HER2) fell short of 100% of cases, but very few patients lacked these data. In contrast, the Ki-67 cell proliferation marker had a significantly lower number of records, making molecular classification and TNM staging impossible.

The various forms of presentation that led to a breast cancer diagnosis in our observation group were also analysed. Table 1 shows that a clinical manifestation led 31% of patients to a consultation. In over 50% of patients, diagnosis followed detection of nodules, incidental findings or suspicious images, showing that clinical screening strategies and mammography make early breast cancer diagnosis possible [6, 7]. Table 3 shows how frequently patients received any type of treatment in the course of their disease, listed by therapeutic group (Hormone therapy, Chemotherapy, Targeted Therapy or Immunotherapy). The most frequently used drugs are also noted for each treatment type. Another finding was that chemotherapy and hormone therapy are the most common breast cancer treatments, followed by targeted therapies. There were very few cases of immunotherapy. Data obtained to assess quality of life showed good treatment tolerance and performance, even in advanced stages of breast cancer (Table 4).

## Discussion

Data on treatment progress for the general population outside of systematised studies has become vital to making appropriate decisions in research, developing new molecules and implementing health policies. The problems in obtaining this data are well known. Disorganised, incomplete databases that are not systematised and make it cumbersome to upload data are one problem. Another is that professionals are unwilling to collaborate with database projects as it adds to their daily workload. The PrecisaXperta platform helps minimise these problems.

The exponential increase in the generation of health data is linked to the development of medical Big Data management tools. Millions of data are generated globally and the utility we derive from them will depend on systematising and organising those data. Interconnectivity related to internet access and virtual storage have been vital to the growth of data. By 2025, it is expected that the medical field will lead the world in developing Big Data [8–10]**.**

The PrecisaXperta platform is an online database with a convenient graphical environment designed so doctors can add RWD from cancer patients quickly and easily. PrecisaXperta facilitates data recording by interconnecting all epidemiologic data to create statistics in real time.

Precision medicine and bioinformatics are generating data while genomic studies are processing information, specifically for clinical oncology. These trends highlight the importance of having reliable, real-world clinical data and building predictive models using AI [11].

### Data processing in PrecisaXperta

PrecisaXperta uses an algorithm to anonymise all data the system records. This algorithm automatically converts personal and identification data into an irreversible 8-digit alphanumeric code. Only the health professional caring for the patient can reverse data anonymisation; using a unique, personal username and password to turn the code back into patient data. The information that PrecisaXperta receives and processes is strictly limited to epidemiological data and does not include sensitive patient data.

To ensure data security and protection, PrecisaXperta encrypts all content and processing using the Advanced Encryption Standard method (more commonly known as Rijndael), which the US government uses to protect general data. Argentine law requires Fundación Investigar to register any database it creates in the National Personal Data Protection Registry (RNPDP in Spanish). The Ministry of Justice oversees the Registro de Protección de Datos Personales (RNDPDP) and issues certifications of databases (under Law No. 25.326 and its implementing regulations approved under decree No. 1558/01). To enhance anonymisation, PrecisaXperta limits geolocation to provinces and does not record cities, addresses or institutions. This extra protection prevents the database from recording information that could be linked to patient identities.

Ethical data management is vital, and preserving privacy, protecting sensitive data and guaranteeing irreversible anonymisation are high priorities in operating PrecisaXperta [12, 13]**.** The benefits of an open, transparent database for participating researchers; the safety and protection of data; and the responsibility for the accuracy of those data are integral to operating PrecisaXperta [12, 14].

Pre-determined programming processes ensure that data uploads are validated. PrecisaXperta then records any subsequent changes, revisions or corrections to data, with the name of the user who makes them and the date and time.

**Figure 2.**
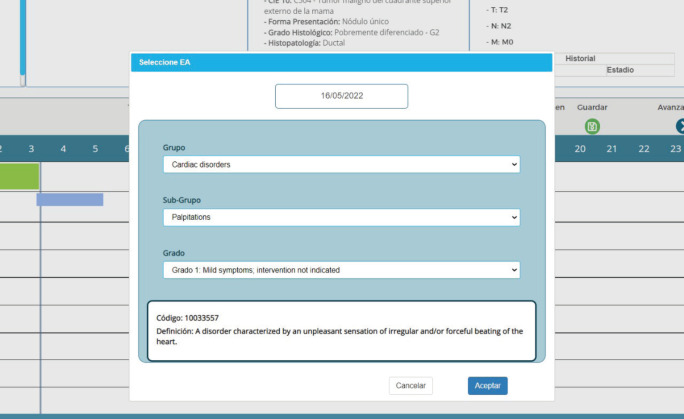
CTCAE v5.0-based adverse event entry in PrecisaXperta.

A user-friendly interface makes data entry easy to master without a learning curve. The upload sequence uses the same logic as the description of a medical case. Figure 2 shows the upload screens for an adverse event, which use the Common Terminology Criteria for Adverse Events, Version 5.0 system. Users can enter this data without typing, making it easy to complete this sequence quickly.

### Data upload algorithm and systemisation

**Figure 3.**
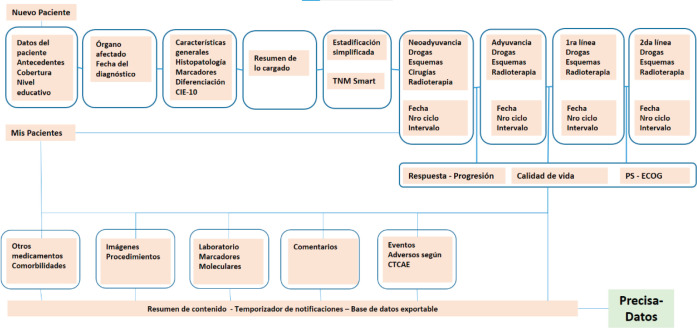
PrecisaXperta site map.

Figure 3 provides a map of PrecisaXperta website contents that is clear and easy to grasp. This map shows the different fields on the platform and the various ways for completing them, which vary in complexity.

An indirect aim of this article is to determine which of the fields among these alternatives are essential for reflecting the epidemiology of the field a study covers. The study found that the patient admission and discharge sequences, history of pre-existing disease, molecular markers for classification and staging should satisfy this requirement.

PrecisaXperta uses an intelligent staging system that makes it possible to calculate stages automatically simply by indicating the T (tumour) N (node) and M (metastasis) status. The platform also provides guidelines from the National Comprehensive Cancer Network when necessary. The intelligent staging system is a plug-in, TNMsmart, which makes this task easy and distinguishes PrecisaXperta as having a higher percentage of staged patients [International Classification of Diseases for Oncology (ICD-O)-O – 50 in the international code] than other databases. From 40% to 50% of tumours in most epidemiological registries lack staging data because it is difficult to obtain [5].

PrecisaXperta periodically undergoes remote monitoring processes. This Food and Drug Administration (FDA)-approved method, reflecting that nearly all research studies use electronic case report forms, asks researchers to send the documentation that supports their data sources digitally. Monitoring the data entered and verifying its validity is essential to ensuring data reliability and transparency [15, 16].

**Figure 4.**
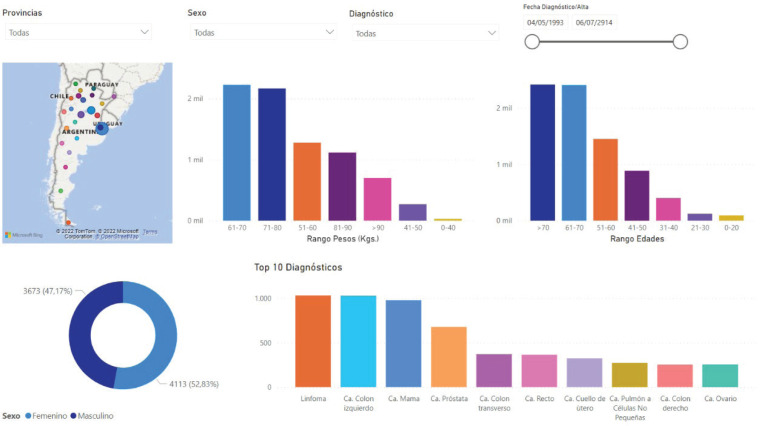
Online statistical graphs in Precisa-Datos.

Figure 4 shows one of the statistics screens the system provides as an example. The platform creates this screen automatically using the anonymised flat data prepared for export to the statistical analysis tools and AI engines. Data updates occur in real time and all researchers using the platform have access to current data. One of the most appealing features that convinces professionals to participate in this project is the information display. Custom filters let users set parameters for viewing clinical-epidemiological data they can use in personal studies and publications.

PrecisaXperta also automatically generates ancillary documents that help users in their professional roles as caregivers and researchers. These documents include summaries of uploads, audit forms and an informed consent to share anonymised personal data that users can print and keep.

**Figure 5.**
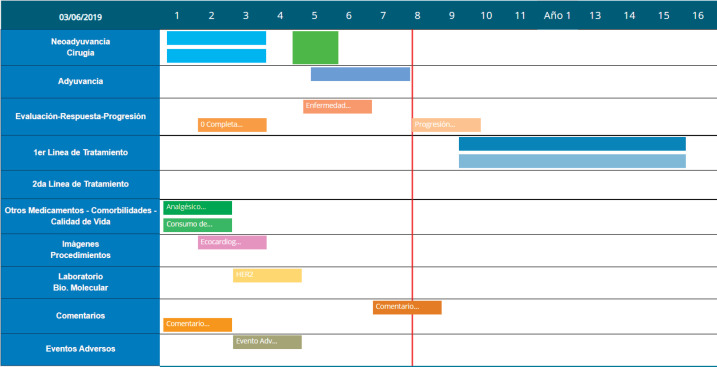
Timeline with patient progress example.

The platform also documents patient progress using a ‘Timeline’ that displays two Cartesian axes. The horizontal axis represents time elapsed and the vertical axis represents the upload options for patient follow-up. Another feature is Smart-labels, icons indicating what data has been uploaded and synchronised to appear on the screen on relevant dates and remind users what data details to save (see Figure 5).

### Quality of breast cancer data obtained

The PrecisaXperta RWD collection platform is configured for any solid tumour. But of the data obtained for 6,892 patients, only those with a breast cancer diagnosis were selected for this analysis. The rationale for this selection was to focus on a condition (the most prevalent in our database) and conduct a descriptive analysis of the data obtained, its quality, validation and inconsistencies. That analysis could make it possible to suggest fundamental improvements in future Big Data management.

The recorded anthropometric data are consistent with the average female population for this age group [17]. The largest group of patients had no history (23.13%), but a significant number of patients were obese (10.88%) and BMI of 25.2 Kg/m^2^ indicated pre-obesity. These findings could lead to future detailed analyses of excess weight and breast cancer in Argentina.

The TNMsmart system, which automatically stages all solid tumours, has ensured that staging is complete for most patients. Some differences were found when stages at diagnosis from the Registro Institucional de Tumores de Argentina Instituto Nacional del Cáncer (RITA-INC) database were compared with data from this descriptive analysis. In PrescisaXperta, 36.48% of patients were in stage I at diagnosis versus 9.9% in RITA-INC. The figures for other stages were: 30.06% versus 22.9% in stage II, 19.61% versus 16.2% in stage III and 7.56% versus 7.1% in stage IV, respectively. These differences could be attributed to a disparity in the numbers of patients staged. They may also reflect that the public health system (the main source of RITA-INC data) treats a greater proportion of advanced cancer cases than the providers who use PrecisaXperta, whose patients have medical insurance [5–7].

Luminal A and B, basal-like, non-basal-like and triple-negative stratification are already routine in breast cancer management. This descriptive analysis found that most cases had hormonal and HER2 markers and a very low percentage had the Ki-67 marker, showing that molecular classification data was incomplete. The descriptive analysis also found that ensuring complete molecular classification requires labelling the molecular marker set mandatory data [18–23].

The distribution of treatment types in the analysed group showed that over 60% of patients received chemotherapies and hormone therapies. The main chemotherapy treatments were Cyclophosphamide, Adriamycin and Paclitaxel, and the main hormone treatments were Trastuzumab, Palbociclib and Pertuzumab. Patients whose molecular markers made them candidates for targeted therapies received them, showing that Argentina offers good access to high-cost breast cancer medications. Comparing data on health coverage and access to these medications found no significant differences between patients covered by the Social Security system, those with health insurance and uninsured hospital patients. But there was little insurance data in this sample [24].

Other interesting data obtained on breast cancer highlight the importance of real-world records, which are generally not objectives of controlled studies [25]. The quality of life assessment shows good medication tolerance, even in the most advanced stages. Pain is a symptom in almost all stages of treatment, albeit with a very low intensity, but patients continued daily activities without help until stage IV. This analysis also confirmed a relationship between a higher level of education and lower staging (I and II) at breast cancer diagnosis, independent of the health coverage for the patient. This finding suggests that the data received through prevention campaigns is more effective for patients with higher educational attainment and is instrumental in early diagnosis in a country with universal access to medical care [24]. Functional predictive algorithm engines that process Big Data require a volume of data that does not yet exist. Until it does, RWD will provide extremely useful, descriptive, population-wide insights when assessing factors related to oncology trends and treatments.

## Conclusion

This is the first RWD collection on patients with cancer which uses a systematised online platform for uploading clinical oncology data. A multidisciplinary team designed the PrecisaXperta platform and created a user-friendly, intuitive graphical interface. The platform records data for patient age, comorbidities, staging, molecular markers, quality of life and types of treatment received. This study offers a limited but promising view of using PrecisaXperta as an integrative platform to collect reliable oncology data. The analysis of the selected disease (breast cancer) to assess data quality shows that some fields should be mandatory. Another finding was that molecular markers (especially Ki-67) and the dates-treatments-control-response sequence should also be considered mandatory criteria. These data should also be collected at least every 90 days to document disease progression thoroughly [26–28]. Fundación Investigar is working to generate epidemiological Big Data, develop AI-based predictive algorithms and make them available to the medical community. The overarching aim of this work is to contribute to effective cancer treatments for patients.

## Conflicts of interest

Dr Guillermo Streich declares no conflicts of interest associated with this publication.

Dr Marcelo Blanco Villalba declares no conflicts of interest associated with this publication.

Mr Christian Cid declares having contributed to the development of this platform at Fundación Investigar.

Dr Guillermo Bramuglia declares being the Scientific Director of Fundación Investigar.

## Funding

Fundación Investigar is a non-profit non-governmental organization that promotes Human Health research. No contributions or funding have been received to conduct this work.

## Figures and Tables

**Table 1.** Description of the sample analysed (1,654).

Anthropometry	Average (SD)
Ages (Years)	55.3 (11.88)
Weight (Kg)	67.8 (11.58)
Height (cm)	163.8 (6.02)
Body surface (m^2^)	1.69 (0.20)
Comorbidities. (no.)	% of total
No. recorded (391)	23.63
Diabetes (241)	14.57
Obesity (180)	10.88
Smoking (146)	8.82
Osteoporosis (123)	7.43
Depression (89)	5.38
Hypertension (85)	5.13
Indigestion (65)	3.92
Asthma (41)	2.47
Other^a^ (293)	17.71
Forms of presentation	
Symptomatic (510)	31%
Single nodule (406)	24.70%
Incidental finding (254)	14.7%
Imaging (212)	12.90%
Biopsy (54)	3.30%
Multiple nodules (35)	2.10%
Surgery (23)	1.40%
Miliary spread (12)	0.70%
Brain metastases (7)	0.40%
Thoracic metastases (5)	0.30%
No data (137)	8.30%

a Those diseases are several conditions that are not related to cancer or degenerative disorders linked to cancer

**Table 2.** Sample tumour characteristics, No: 1,654.

Staging (No.)	% of total
0 – in situ (177)	6.03
IA (157)	13.4
IB (281)	23.34
IIA (278)	22.92
IIB (85)	7.14
IIIA (98)	8.14
IIIB (120)	9.97
IIIC (18)	1.5
IV (91)	7.56
Hormone receptors (No.)	% of total
HR positive (1,424)	89.96%
HR negative (159)	10.04%
HER2 receptors (No.)	% of total
HER2 positive (278)	19%
HER2 negative (1209)	81.30%
Cell proliferation receptors (No.)	% of total
Cell proliferation marker Ki-67 positive (216)	13.60%
Cell proliferation marker Ki-67 negative (135)	8.16%
Cell proliferation marker without data (1,303)	78.78%

**Table 3.** Patients by treatment received^a^ No: 1,654.

Treatments	No. of patients	% of total	Most used drugs
Hormone therapy	1,018	61.5	Tamoxifen; Letrozole; Anastrozole
Chemotherapy	1,242	75.9	Cyclophosphamide; Paclitaxel; Adriamycin
Targeted therapy	521	31.4	Trastuzumab; Palbociclib; Pertuzumab
Immunotherapy	6	0.36	Atezolizumab

(a) Received at any point in their progression, without distinguishing treatment stages

**Table 4.** Quality of life assessment by stage.

Items considered	IA–IB	IIA–IIB	IIIA–IIIB	IV
Full daily living activities	95%	96%	70%	20%
Limited daily living activities	5%	4%	20%	65%
Pain free	70%	75%	60%	0%
Non-disabling pain	30%	25%	60%	No data
Full mobility	95%	95%	92%	15%
Limited mobility	5%	5%	8%	98%
